# Effects of prone versus supine position during enteral nutrition on clinical outcomes and complications in mechanically ventilated patients: a systematic review and meta-analysis

**DOI:** 10.3389/fnut.2025.1685664

**Published:** 2026-01-08

**Authors:** Mei-Ling Zhao, Yun-Feng Hou, Li-Wu Zong, Yi-Feng Yue, Shuang Ma

**Affiliations:** 1 Department of Critical Care Medicine, Zibo Central Hospital, Zibo, Shandong, China; 2 Department of Critical Care Medicine, The First Affiliated Hospital of Shandong First Medical University, Jinan, Shandong, China; 3 Department of Anesthesiology, Zibo Central Hospital, Zibo, Shandong, China

**Keywords:** enteral nutrition, prone position, supine position, mechanical ventilation, ventilator-associated pneumonia, gastric residual volume

## Abstract

**Background:**

Enteral nutrition (EN) is routinely employed in mechanically ventilated patients, including those undergoing prone positioning for severe respiratory failure. However, the impact of body positioning during EN on clinical and nutritional outcomes remains unclear. This systematic review and meta-analysis aimed to evaluate the effects of prone versus supine positioning during EN on caloric intake and related complications.

**Methods:**

A comprehensive literature search was conducted in PubMed, The Cochrane Library, Web of Science, Embase, WanFang Data, CNKI, and SinoMed through June 16, 2025. Eligible studies included randomized controlled trials and cohort studies comparing EN outcomes between prone and supine positioning in adult mechanically ventilated patients. Data extraction, quality assessment (Newcastle–Ottawa Scale), and meta-analyses were performed in accordance with PRISMA guidelines.

**Results:**

Five studies comprising 319 patients were included. Prone positioning was associated with significantly lower caloric intake (SMD = −1.31, 95% CI: −2.43 to −0.18), higher odds of vomiting (OR = 2.31, 95% CI: 1.63–3.26), high gastric residual volume (OR = 2.72, 95% CI: 1.47–5.03), ventilator-associated pneumonia (OR = 2.31, 95% CI: 1.34–3.99), and EN interruption (OR = 4.75, 95% CI: 2.22–10.17). No significant difference in mortality was observed (OR = 1.11, 95% CI: 0.65–1.88). No publication bias was detected.

**Conclusion:**

Compared to the supine position, EN during prone positioning is associated with reduced nutritional intake and increased gastrointestinal and pulmonary complications. Optimized feeding protocols are warranted to ensure safe and effective nutritional support in prone-ventilated patients.

## 1 Introduction

Enteral nutrition (EN) constitutes a cornerstone of supportive care in mechanically ventilated patients, aiming to preserve gut mucosal integrity, attenuate systemic inflammation, and prevent infectious complications (1). Adequate EN delivery is essential for the recovery of critically ill patients, as it provides the necessary nutrients to maintain organ function, support immune responses, and prevent malnutrition (2, 3). However, optimal delivery of EN requires careful consideration of multiple factors, including patient positioning, gastric motility, and aspiration risk (4). The prone position has been increasingly adopted in the management of acute respiratory distress syndrome (ARDS) to improve oxygenation and ventilator synchrony, with studies demonstrating its benefits in enhancing pulmonary recruitment and reducing the work of breathing (5). Despite these benefits, concerns persist regarding the impact of prone positioning on EN tolerance, nutrient delivery, and the incidence of gastrointestinal and pulmonary complications. The alteration in gastric physiology and increased intra-abdominal pressure associated with prone positioning may affect gastric emptying, leading to feeding intolerance (6). Conversely, supine positioning, which represents the traditional approach for EN administration, is more familiar and widely practiced; however, it may compromise pulmonary mechanics, particularly in patients with severe hypoxemia, and increase the risk of ventilator-associated pneumonia (VAP) due to ineffective clearance of secretions (7, 8).

Mechanically ventilated patients often experience delayed gastric emptying and impaired gastrointestinal perfusion, which are exacerbated by the use of sedative agents, catecholamine support, and the inflammatory milieu (9, 10). These physiological alterations increase the risk of feeding intolerance, a condition characterized by high gastric residual volumes, vomiting, and regurgitation, which can lead to aspiration and subsequent pneumonia. Studies have shown that feeding intolerance in mechanically ventilated patients is linked to worse clinical outcomes, including increased length of stay, prolonged mechanical ventilation, and higher mortality rates (11, 12). Several observational studies have suggested that prone positioning exacerbates feeding intolerance by altering intra-abdominal pressure and gastric emptying dynamics, which can further impair the gastrointestinal tract’s ability to tolerate enteral feeds (6). However, few studies have comprehensively compared both clinical and nutritional outcomes, particularly in the context of mechanically ventilated adult patients (13, 14). While prone positioning is frequently employed for managing severe respiratory failure, its effects on nutritional delivery and gastrointestinal tolerance are not well understood (15). This lack of a comprehensive comparison creates a gap in the literature, particularly regarding the combined impact on nutritional outcomes (e.g., caloric intake, gastric residual volume) and clinical complications (e.g., VAP, mortality) in patients receiving EN (16, 17).

In this systematic review and meta-analysis, we aimed to address this gap by evaluating the effects of prone versus supine positioning during EN on both clinical outcomes and nutritional parameters in mechanically ventilated adult patients. By integrating data from randomized controlled trials and observational cohort studies, this analysis seeks to clarify the balance of risks and benefits associated with EN administration in these positions. The findings are anticipated to inform clinical decision-making and guide the development of optimized nutritional support strategies for mechanically ventilated patients requiring prone positioning.

## 2 Methods

### 2.1 Search strategy

The literature search was performed in accordance with the Preferred Reporting Items for Systematic Reviews and Meta-Analyses (PRISMA) statement (18). We queried PubMed, The Cochrane Library, Web of Science, Embase, WanFang Data, CNKI, and SinoMed from database inception through June 16, 2025, without language restrictions; non-English articles were included if an English abstract was available. Eligible study designs comprised randomized controlled trials and cohort studies comparing prone versus supine positioning during EN in mechanically ventilated adult patients. Controlled vocabulary (e.g., MeSH terms in PubMed and Emtree in Embase) and free-text words were combined to capture four core concepts: enteral nutrition (e.g., “Enteral Nutrition” OR “tube feeding” OR “gastrointestinal feeding”), prone position (e.g., “Prone Position” OR “ventral decubitus”), supine position (e.g., “Supine Position” OR “dorsal decubitus”) and mechanical ventilation (e.g., “Respiration, Artificial” OR “mechanical ventilation” OR “ventilated patients”). Boolean operators were used to join synonyms and variant spellings, ensuring comprehensive retrieval. Search strategies were tailored to each database’s indexing and syntax but retained identical thematic blocks; the detailed PubMed query is presented in Supplementary Table 1.

### 2.2 Inclusion criteria and exclusion criteria

Studies were considered eligible for inclusion if they met the following criteria:

1) Population: Adult patients (≥18 years) who were receiving invasive mechanical ventilation in an intensive care unit (ICU) or similar critical care setting.
2) Intervention: EN administered while patients were positioned in the prone position.
3) Comparison: EN administered while patients were positioned in the supine position.
4) Outcomes: Studies that reported at least one of the following clinical or nutritional outcomes: (1) Nutritional delivery parameters (e.g., mean caloric intake, gastric residual volume, feeding interruptions); (2) Clinical outcomes (e.g., incidence of VAP, mortality).

Studies were excluded if they met any of the following criteria:

1) Population: Studies involving pediatric patients (<18 years), non-ventilated patients, or animal subjects.
2) Intervention: Studies in which EN was not clearly administered during prone or supine positioning, or in which positioning was not explicitly compared.
3) Outcomes: Studies that did not report any relevant clinical or nutritional outcome data.
4) Study Design: Case reports, case series (*n* < 10), conference abstracts, letters, editorials, reviews, and study protocols.
5) Duplication: Duplicate publications, unless reporting unique data not included in the primary study.

### 2.3 Data extraction

Data extraction was performed independently by two reviewers based on the predefined inclusion and exclusion criteria. Titles and abstracts of all retrieved studies were initially screened to exclude irrelevant articles. The remaining studies were assessed in full text to determine final eligibility. Data from eligible studies were extracted using a standardized form, and any discrepancies between the two reviewers were resolved through discussion or consultation with a third reviewer. The extracted information included the first author, year of publication, underlying disease condition, details of the intervention and control groups, nutritional support plan, strategies for managing EN intolerance, reported clinical outcomes, and high gastric residual volume (HGR) data. All extracted data were cross-checked for consistency and accuracy.

### 2.4 Quality assessment

The risk of bias of the included studies was independently assessed by two reviewers using standardized tools. All assessments were cross-checked, and any disagreements were resolved through discussion and consensus between the two reviewers. For cohort studies, the Newcastle–Ottawa Scale (NOS) was employed to evaluate methodological quality (19). The NOS was independently performed by the same two reviewers who selected the publications. After completing the screening and data extraction process, the reviewers assessed the risk of bias and methodological quality using NOS. Any discrepancies in the assessment were resolved through discussion and consensus. The NOS evaluates studies across three key domains: selection of study groups, comparability of groups, and assessment of outcomes. The total score ranges from 0 to 9, with higher scores indicating better methodological quality. Studies with a score of 7 or above were considered high quality, while those scoring below 7 were considered moderate or low quality.

### 2.5 Statistical analyses

All statistical analyses were conducted in accordance with standard meta-analytic procedures using Stata version 18.0. For dichotomous outcomes, pooled effect estimates were expressed as risk ratios (ORs) with corresponding 95% confidence intervals (CIs). For continuous outcomes, standardized mean differences (SMDs) with 95% CIs were calculated depending on the consistency of measurement scales across studies. Heterogeneity across studies was assessed using the Chi-squared (*χ*^2^) test and quantified with the *I*^2^ statistic. An *I*^2^ value >50% and/or *p* < 0.10 indicated substantial heterogeneity, in which case a random-effects model (DerSimonian–Laird method) was applied. Otherwise, a fixed-effects model (Mantel–Haenszel method) was used. Egger’s regression test and Begg’s rank correlation test were applied to detect small-study effects. A *p*-value < 0.05 was considered statistically significant for all tests, unless otherwise specified.

## 3 Results

### 3.1 Search results and study selection

**Figure 1:**
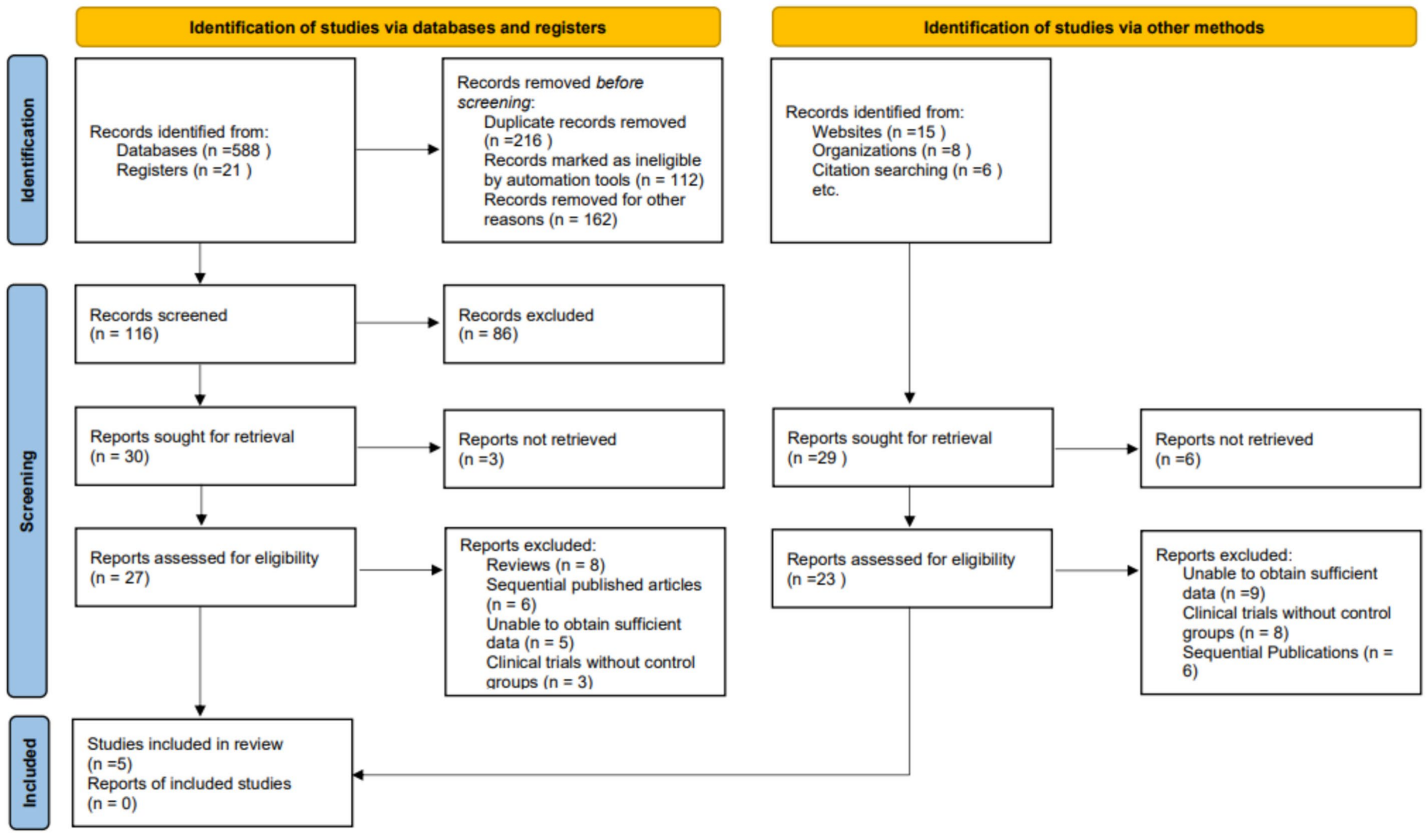
PRISMA flow diagram showing the process of study selection for the systematic review and meta-analysis.

A total of 609 records were identified from database and registry searches (588 from electronic databases, 21 from trial registers). An additional 29 records were found through other sources (websites, *n* = 15; organizations, *n* = 8; citation searches, *n* = 6). After removing 216 duplicates and excluding 277 records (112 ineligible via automation, 165 irrelevant), 116 records remained for screening. Following title and abstract review, 86 records were excluded, and 30 full-text articles were retrieved for further evaluation. Of these, 3 reports were not retrievable. A total of 27 full-text articles were assessed for eligibility. Nineteen studies were excluded for the following reasons: review articles (*n* = 8), sequential publications (*n* = 6), insufficient data (*n* = 5), and lack of control group (*n* = 3). An additional 29 records from other sources were sought for retrieval, of which 6 were not retrieved. Of the 23 reports assessed, 9 were excluded due to insufficient data, 8 for lacking control groups, and 6 as sequential publications. Ultimately, 5 studies met the inclusion criteria and were included in the final systematic review and meta-analysis (13, 20–23) (Figure 1).

### 3.2 Study characteristics

This meta-analysis included five prospective studies conducted between 2001 and 2022, encompassing a total of 319 mechanically ventilated adult patients receiving EN in either the prone or supine position. The studies involved diverse clinical populations, including patients with COVID-19, severe hypoxemia, and community-acquired pneumonia (CAP), with illness severity commonly assessed by APACHE II scores. Study designs comprised four prospective observational studies and one prospective crossover study. Positioning protocols varied, with some studies alternating prone and supine positioning every 6 h, while others used fixed or comparative positioning over multiple days. All studies implemented early EN, typically via nasogastric tube, with caloric targets ranging from 15 to 25 kcal/kg/day. Nutritional delivery strategies included gradual increases in infusion rates or stepwise progression in volume. Management of EN intolerance primarily relied on gastric residual volume (GRV) monitoring, with predefined GRV thresholds ranging from >0.15 L to >0.5 L. Feeding was withheld in the presence of excessive GRV, vomiting, or regurgitation, and prokinetic agents were used in some studies to support feeding tolerance (Table 1).

**Table 1:** Summary of study characteristics and enteral nutrition practices in prone vs. supine position during mechanical ventilation.

Study (Year)	Design	Population and condition	Positioning protocol	Enteral nutrition strategy	Management of EN intolerance	HGR threshold	Design
Alves de Paula et al. (2022), (13)	Prospective observational study	126 COVID-19 patients receiving EN on MV	PP with 6-h turns; gastric volume monitored	EN target: 15–20 kcal/kg/day in first week; increased in rehabilitation phase	Adjust EN rates and prokinetics as needed; stop feeding based on GRV; continuous monitoring	Not reported	Prospective observational study
Saez de la Fuente et al. (2016), (22)	Prospective crossover study	34 critically ill ICU patients; 60% APACHE II > 15; most diagnosed with CAP	PP for severe hypoxemia, compared to SP in same patient	EN via NG tube targeting 25 kcal/kg/day; PN used if EN insufficient	Stop EN if GRV > 0.5 L, vomiting, or regurgitation; reassess and resume feeding after correction	>0.5 L (no count)	Prospective crossover study
Songyan et al. (2006) (37)	Prospective observational study	69 patients on MV (34 prone, 35 supine)	Fixed position (PP or SP) for 5 days	EN from 0.03 L/h on Day 1, increased to 0.12 L/h by Day 4–5	GRV monitored daily; EN paused if GRV > 0.15 L; prokinetic agents used	>0.15 L	Prospective observational study
Reignier et al. (2004), (20)	Prospective observational study	71 ventilated patients receiving early EN for 5 days	Prone every 6 h in same patient	EN increased from 0.5 L (Day 1) to 2 L (Day 5); no PPI used	Stop EN if GRV > 0.25 L or vomiting; gastric volume monitored every 6 h	>0.25 L	Prospective observational study
Van der Voort et al. (2001), (21)	Prospective observational study	19 patients; APACHE II: 25.5 ± 8.9	Alternated every 6 h between prone and supine	Continuous EN at 0.08 L/h	10 patients pretreated with cisapride for GRV; EN continued despite high GRV	>0.15 L	Prospective observational study

EN, Enteral Nutrition; MV, Mechanical Ventilation; PP, Prone Position; SP, Supine Position; GRV, Gastric Residual Volume; PPI, Proton Pump Inhibitor; VAP, Ventilator-Associated Pneumonia; PN, Parenteral Nutrition; CAP, Community-Acquired Pneumonia; NG, Nasogastric.

### 3.3 Results of quality assessment

The methodological quality of the included cohort studies was evaluated using the Newcastle–Ottawa Scale (NOS), which assesses three domains: selection of study groups, comparability of cohorts, and outcome assessment. All five studies demonstrated high methodological quality, with total scores ranging from 8 to 9 out of a maximum of 9 points. All studies achieved full scores in domains related to representativeness of the exposed cohort, ascertainment of exposure, and adequacy of follow-up. Most studies also clearly demonstrated that the outcome of interest was not present at the start of the study and provided adequate follow-up duration. Four of the five studies achieved the maximum score of 9, while one study received a score of 8 due to slightly lower comparability of cohorts. Overall, the included studies were judged to have a low risk of bias and were deemed methodologically robust for inclusion in the meta-analysis (Table 2).

**Table 2:** The quality assessment according to Newcastle-Ottawa scale of each cohort study.

Study	Representativeness of the exposed cohort	Selection of the non-exposed cohort	Ascertainment of exposure	Demonstration that outcome of interest was not present at start of study	Comparability of cohorts on the basis of the design or analysis	Assessment of outcome	Was follow-up long enough	Adequacy of follow up of cohorts	Total score
Alves de Paula et al. (2022), (13)	★	★	★	★	★★	★	★	★	9
Saez de la Fuente et al. (2016), (22)	★	★	★	★	★	★	★	★	8
Songyan et al. (2006) (37)	★	★	★	★	★★	★	★	★	9
Reignier et al. (2004), (20)	★	★	★	★	★★	★	★	★	9
Van der Voort et al. (2001), (21)	★	★	★	★	★★	★	★	★	9

★, each individual asterisk (‘★’) signifies one point.

### 3.4 Comparison of mean caloric intake between prone and supine positions

**Figure 2:**
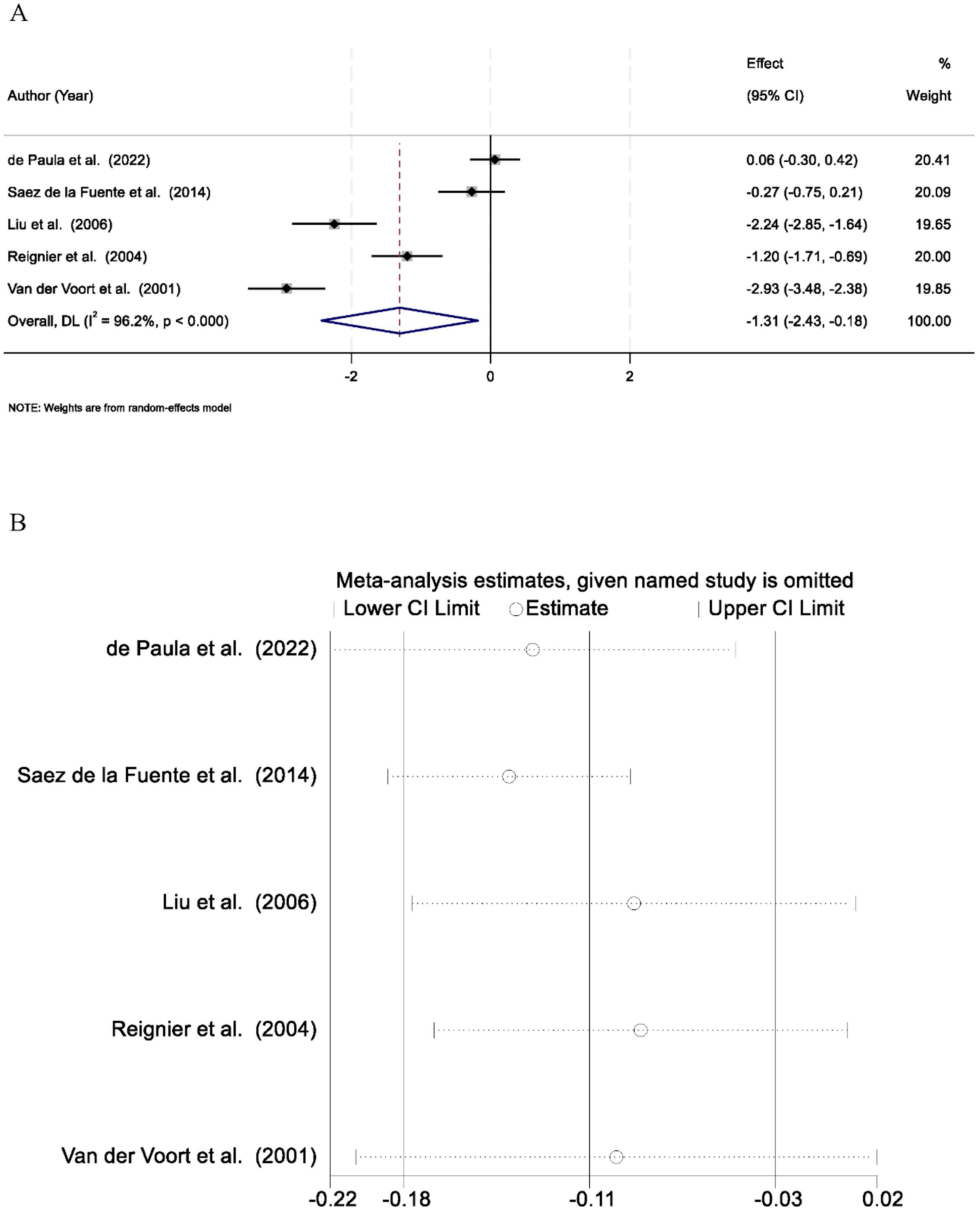
**(A)** Forest plot comparing mean caloric intake between prone and supine positions. Patients in the prone position received significantly less caloric intake (SMD = −1.31, 95% CI: −2.43 to −0.18; *p* < 0.05). **(B)** Sensitivity analysis of caloric intake using the leave-one-out method. The overall result remained stable, indicating no single study significantly influenced the findings.

A total of five studies were included in the meta-analysis to compare mean caloric intake between patients receiving EN in the prone versus the supine position during mechanical ventilation. Significant heterogeneity was observed among the included studies (*I*^2^ = 96.2%, *p* < 0.001), suggesting substantial variability in study populations, nutritional protocols, or clinical settings. Therefore, a random-effects model was applied to account for between-study variability. The pooled analysis demonstrated that patients in the prone position received significantly lower mean caloric intake compared to those in the supine position, with a SMD of −1.31 (95% confidence interval [CI]: −2.43 to −0.18; *p* < 0.05) (Figure 2A). To evaluate the robustness and reliability of the pooled estimate, a leave-one-out sensitivity analysis was performed. The results remained stable across all iterations, indicating that no single study disproportionately influenced the overall effect size (Figure 2B).

### 3.5 Incidence of ventilator-associated pneumonia in prone versus supine position

**Figure 3:**
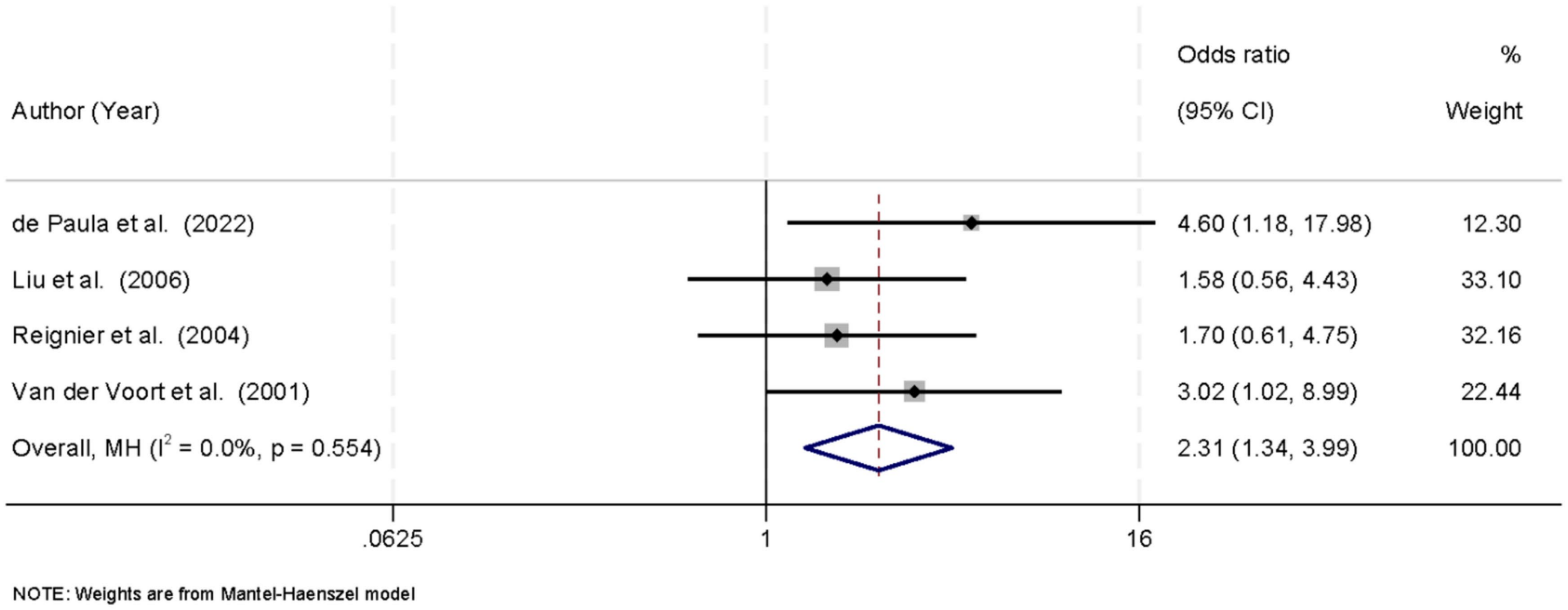
Forest plot of ventilator-associated pneumonia (VAP) incidence. Prone position was associated with a higher VAP risk (OR = 2.31, 95% CI: 1.34–3.99; *p* < 0.05).

Four studies reported data on the incidence of VAP. No significant heterogeneity was observed among the studies (*I*^2^ = 0.0%, *p* = 0.554), and a fixed-effects model was applied. The pooled analysis showed that prone positioning was associated with a significantly higher incidence of VAP compared to the supine position (OR = 2.31, 95% CI: 1.34–3.99, *p* < 0.05) (Figure 3).

### 3.6 Incidence of vomiting in prone versus supine position

**Figure 4:**
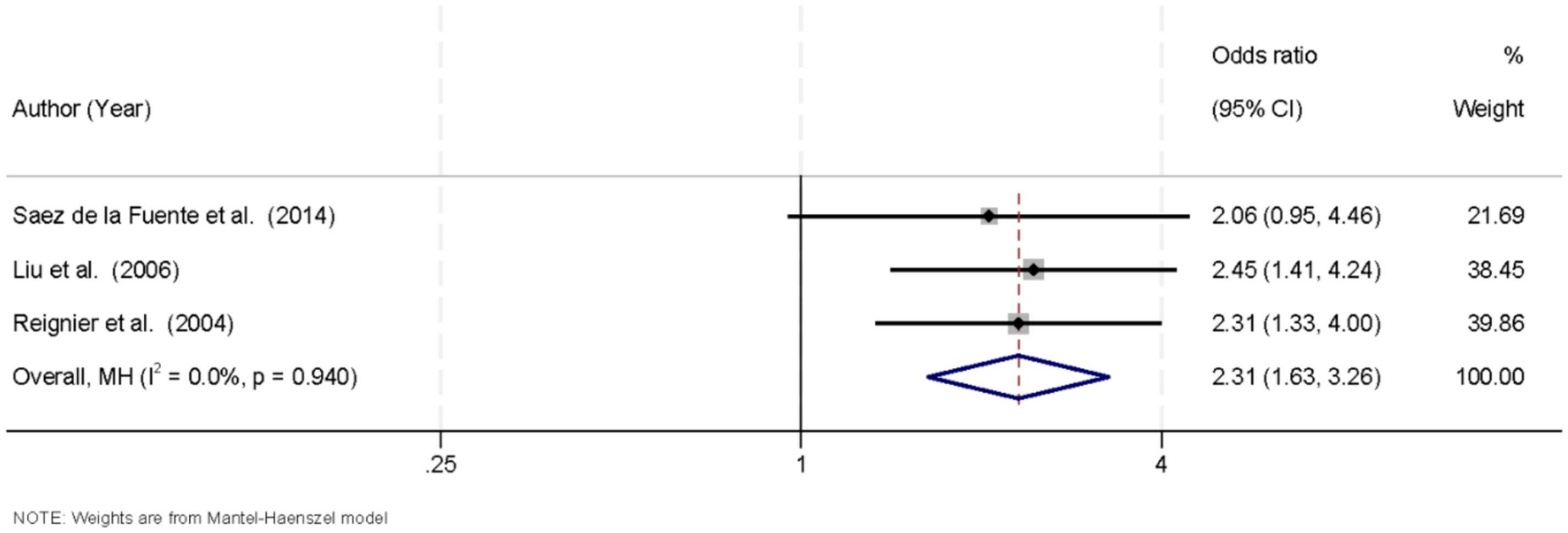
Forest plot of vomiting incidence. Prone positioning significantly increased the risk of vomiting (OR = 2.31, 95% CI: 1.63–3.26; *p* < 0.05).

Three studies were included to assess the incidence of vomiting in patients receiving EN while in the prone or supine position. The heterogeneity among studies was negligible (*I*^2^ = 0.0%, *p* = 0.940), indicating high consistency across the findings. Therefore, a fixed-effects model was applied to pool the results. The meta-analysis showed that the prone position was significantly associated with a higher incidence of vomiting compared to the supine position, with a pooled OR of 2.31 (95% CI, 1.63–3.26; *p* < 0.05) (Figure 4).

### 3.7 Comparison of mortality between prone and supine positions

**Figure 5:**
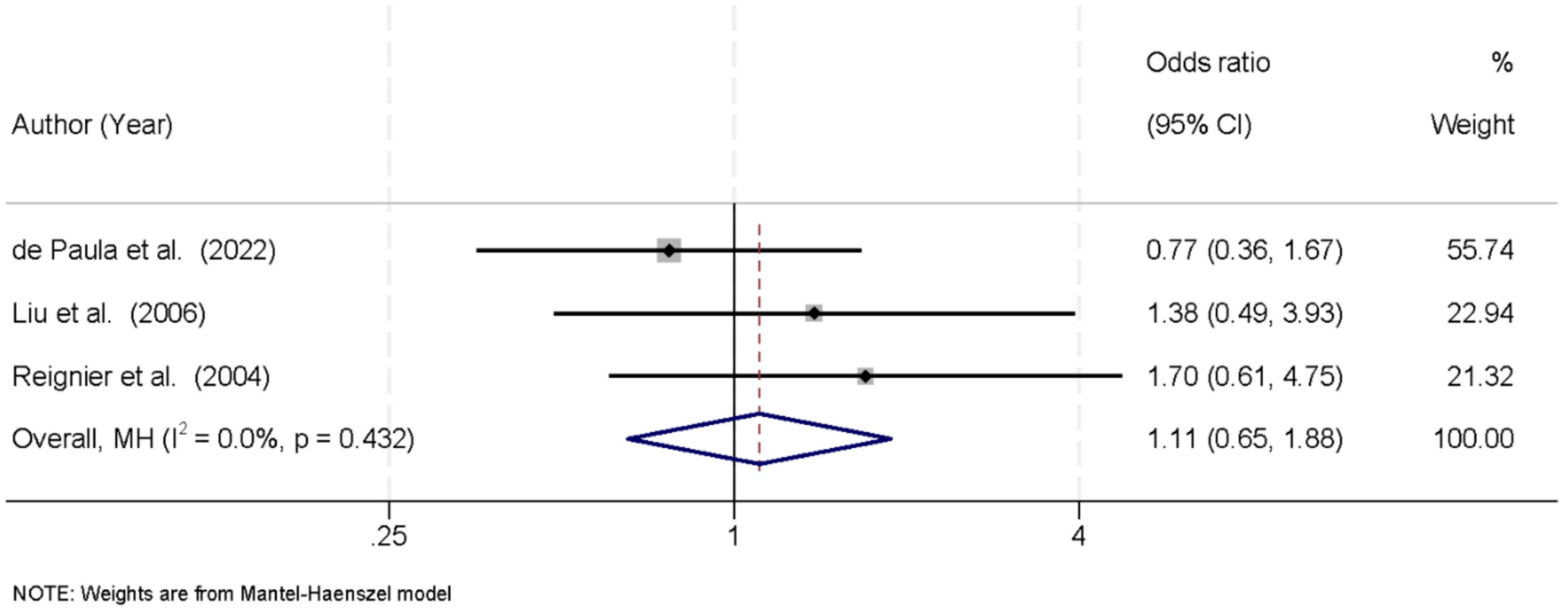
Forest plot of mortality. No significant difference in mortality between prone and supine groups (OR = 1.11, 95% CI: 0.65–1.88; *p* > 0.05).

Three studies reported data on patient mortality associated with EN during prone versus supine positioning. No significant heterogeneity was observed among the studies (*I*^2^ = 0.0%, *p* = 0.432), supporting the use of a fixed-effects model for pooled analysis. The meta-analysis revealed no statistically significant difference in mortality between the prone and supine groups. The combined OR was 1.11 (95% CI: 0.65–1.88; *p* > 0.05) (Figure 5).

### 3.8 Incidence of high gastric residual volume

**Figure 6:**
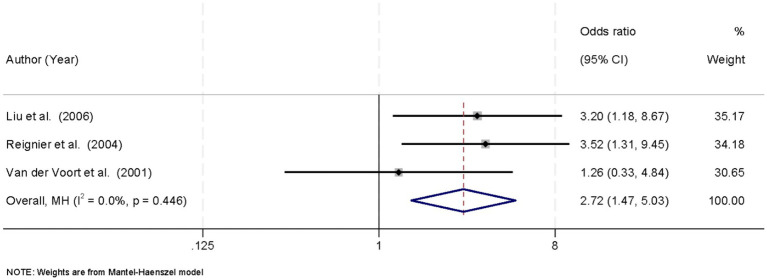
Forest plot of high gastric residual volume (HGRV) incidence. Prone position showed a higher risk of HGRV (OR = 2.72, 95% CI: 1.47–5.03; *p* < 0.05).

Three studies reported the incidence of HGRV in patients receiving EN in the prone versus supine position. No significant heterogeneity was detected across studies (*I*^2^ = 0.0%, *p* = 0.446), supporting the use of a fixed-effects model for data synthesis. The pooled analysis demonstrated that prone positioning was significantly associated with a higher incidence of HGRV compared to the supine position, with an OR of 2.72 (95% CI: 1.47–5.03; *p* < 0.05) (Figure 6).

### 3.9 Incidence of enteral nutrition interruption

**Figure 7:**
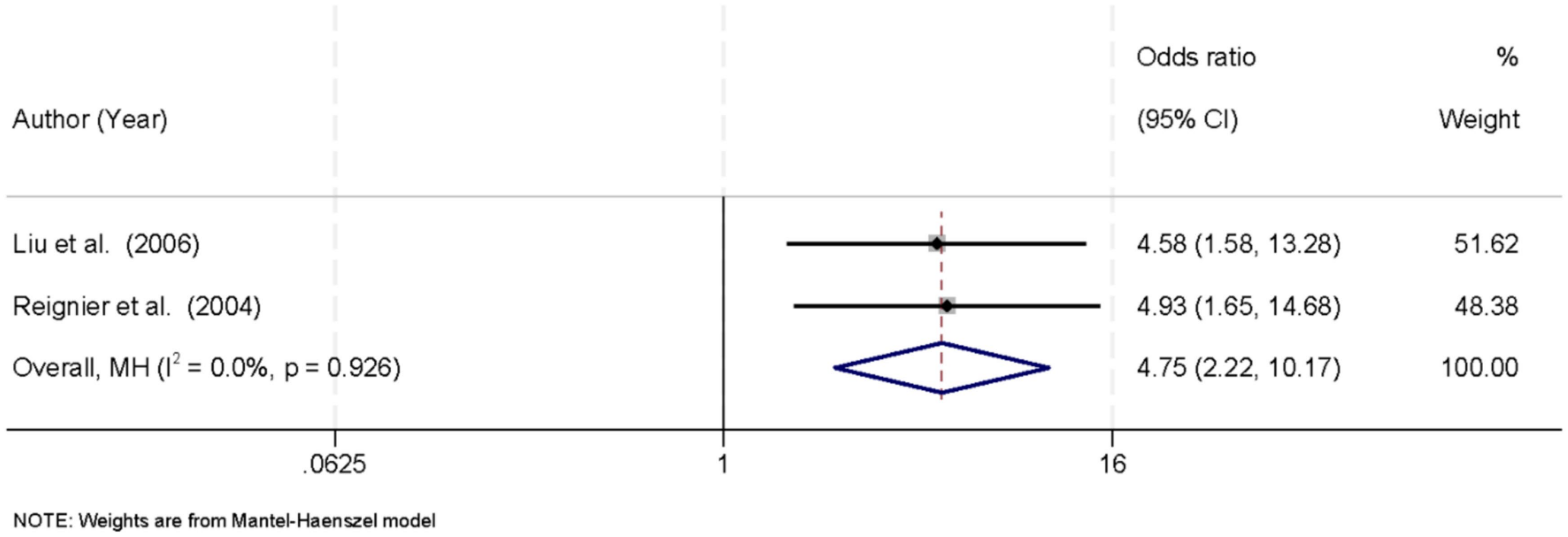
Forest plot of enteral nutrition interruption. EN interruption was more common in the prone group (OR = 4.75, 95% CI: 2.22–10.17; *p* < 0.05).

Two studies reported the incidence of EN interruption in prone versus supine positioned patients. No significant heterogeneity was found (*I*^2^ = 0.0%, *p* = 0.926), and a fixed-effects model was used. The pooled analysis showed that EN interruption was significantly more frequent in the prone position, with an OR of 4.75 (95% CI: 2.22–10.17; *p* < 0.05) (Figure 7).

### 3.10 Publication bias assessment

Assessment of publication bias was conducted using Egger’s linear regression test across all included outcome variables. The results revealed no statistically significant evidence of small-study effects or publication bias (all *p* > 0.05), suggesting that the findings of the meta-analysis are unlikely to be influenced by selective reporting.

## 4 Discussion

This systematic review and meta-analysis demonstrates that EN during prone positioning in mechanically ventilated patients is associated with important nutritional and clinical consequences. Patients fed in the prone position received significantly lower caloric intake on average than those fed in the supine position. This finding suggests that prone positioning can impede adequate nutritional delivery, likely due to more frequent feeding interruptions and intolerance. Notably, a recent large cohort study on COVID-19 patients found that on days patients were proned, fewer calories were administered and undernutrition was more common, whereas supine days achieved higher caloric delivery (6, 24). The reduced energy intake in prone positioning is clinically relevant because cumulative energy deficits can worsen patient outcomes through malnutrition and weakened immunity.

Our analysis also indicates that prone positioning is linked to increased gastrointestinal intolerance. The prone group had a significantly higher incidence of HGRV and vomiting compared to the supine group. These results align with early observations by Reignier et al., who reported that critically ill patients in prone position experienced substantially greater gastric residual volumes on multiple days and more frequent vomiting episodes than those kept supine (25, 26). In that 2004 study, EN had to be halted in 82% of prone-positioned patients (versus 49% in supine) due to intolerance, resulting in lower overall feed volumes (20). Such findings underscore that prone positioning can compromise gastric emptying and increase the risk of regurgitation. The likely mechanisms include elevated intra-abdominal pressure and the need for deep sedation and neuromuscular blockade in prone ventilation, which together slow gastrointestinal motility and blunt protective reflexes. In contrast, some later studies did not observe significant differences in feeding tolerance between positions (27). For example, a prospective study by Saez de la Fuente et al. found no significant difference in daily gastric residuals or vomiting frequency when comparing prone versus supine feeding, suggesting that EN was feasible and safe in prone patients under their protocol (22). Those contradictory findings may reflect improvements in care (such as routine use of prokinetic agents, post-pyloric feeding tubes, and careful positioning) that mitigated intolerance in prone-fed patients. Indeed, a 2020 systematic review noted that while several studies reported no difference in gastric residual volumes between prone and supine positions, at least one study did find higher residuals during prone feeding and another found the opposite. Similarly, evidence on vomiting has been mixed, with one study reporting more frequent vomiting in prone position and another showing no significant difference (28). Our pooled results, drawn from a larger aggregated sample, help resolve these inconsistencies by demonstrating a clear overall trend of reduced EN tolerance associated with prone positioning.

Perhaps most importantly, our meta-analysis reveals that prone positioning during EN is associated with a significantly higher risk of VAP. Patients fed enterally while prone had over twice the odds of developing VAP compared to those fed supine. This finding is noteworthy because previous data on aspiration pneumonia risk in prone positioning have been limited. In one earlier study included in a prior review, the incidence of aspiration pneumonia did not differ between prone and supine groups. By contrast, our analysis pooling four studies detected a significant increase in VAP with prone positioning. The higher VAP incidence could be a consequence of the increased vomiting and regurgitation in prone patients, leading to a greater chance of gastric contents being aspirated into the lungs (29). Additionally, prone positioning often necessitates a flat or head-down posture (since maintaining head-of-bed elevation is challenging in the prone position), which may facilitate aspiration of secretions. The need for heavy sedation and paralysis to tolerate proning might further impair airway protective reflexes, compounding the aspiration risk. Our findings therefore highlight a previously underappreciated trade-off: while prone positioning can improve oxygenation in ARDS, it may simultaneously elevate the risk of VAP when patients are being enterally fed (30, 31). Encouragingly, we found no statistically significant difference in mortality between prone and supine groups. This suggests that the nutritional deficits and higher complication rates associated with prone positioning did not translate into an observable increase in short-term mortality, although the number of studies and patients may be insufficient to detect a small mortality effect. It is also possible that the benefits of prone positioning on respiratory function counterbalance its nutritional drawbacks, resulting in neutral overall survival outcomes. This is consistent with prior observations that, in at least one study, mortality did not differ with prone feeding versus supine (28). Nonetheless, avoidance of complications like VAP remains crucial, as they can prolong ICU stay and morbidity even if mortality is unaffected.

In the context of existing literature, our results both corroborate and extend prior knowledge. Earlier investigations into EN during prone positioning were few and yielded mixed results. A systematic review in 2020 concluded that the evidence was sparse and sometimes contradictory regarding gastric residuals and complications in prone-fed patients. Our analysis, which incorporates several recent studies (including data from the COVID-19 pandemic when prone ventilation became more widespread), provides a more definitive assessment. We confirm the concerns raised by Reignier et al. about poor tolerance of gastric feeding in prone patients, while providing quantitative estimates of risk increases (e.g., roughly 2.3-fold higher odds of vomiting) that were not previously available (20). At the same time, our findings challenge the notion promoted by some subsequent studies that prone positioning has no adverse effect on nutrition tolerance (20). Instead, it appears that without meticulous management, prone positioning can significantly compromise nutritional intake and safety. Differences in findings across studies likely reflect variations in feeding protocols. Centers that reported no differences often employed measures such as semi-recumbent prone positioning, prophylactic prokinetics, or post-pyloric feeding tubes, which can reduce gastric stasis and aspiration. By contrast, studies reporting large deficits and complications often allowed unrestricted gastric feeding in fully pronated, flat patients, thereby exposing the full extent of positional impact (32, 33). Our meta-analysis synthesizes these varying practices, and the substantial heterogeneity observed in caloric intake outcomes (*I*^2^ ≈ 96%) indeed suggests that practice variation was large. Overall, the weight of evidence now indicates prone positioning poses challenges to enteral feeding that clinicians must proactively address.

These findings carry several important clinical implications for the care of mechanically ventilated patients. First, clinicians should be aware that patients in the prone position are at high risk for insufficient caloric delivery. Careful strategies are needed to ensure nutritional goals are met despite prone positioning – for example, using higher calorie-density feeds or supplemental par EN if enteral feeding is frequently interrupted. Second, the significantly increased risks of vomiting and VAP in proned patients highlight the need for vigilant preventive measures (34, 35). Implementing semi-recumbent positioning (tilting the bed to elevate the head and chest even while prone), using prokinetic agents to promote gastric emptying, and considering post-pyloric (nasojejunal) feeding tubes can all mitigate aspiration risk. Frequent monitoring of gastric residuals and signs of intolerance is essential; some protocols suggest reducing or pausing feeds when patients are turned prone, then resuming cautiously once stabilized. Current critical care nutrition guidelines do state that prone positioning is not an absolute contraindication to enteral feeding, and our results support continuing EN in prone patients with appropriate safeguards. In practice, a balanced approach is warranted – one that continues the proven benefits of early EN for critically ill patients, but adapts feeding techniques to minimize the added risks introduced by the prone posture (28, 36).

This study presents several notable strengths. It represents one of the most comprehensive quantitative syntheses to date on the effects of prone versus supine positioning during EN in critically ill patients. By integrating data across multiple studies, this meta-analysis enhances statistical power and enables a broader evaluation of clinical outcomes, including nutritional intake, gastrointestinal tolerance, and complications such as VAP and mortality. However, several limitations warrant consideration. Most included studies were observational, increasing the potential for confounding due to disease severity or other unmeasured variables. Additionally, variation in enteral feeding protocols—such as timing, feeding route, and use of prokinetic agents—likely contributed to outcome heterogeneity, especially in caloric intake. The limited number of studies for some endpoints (e.g., vomiting, high gastric residual volume) reduces the precision and may limit the strength of subgroup conclusions. Moreover, inconsistent VAP definitions across studies may have introduced misclassification bias. Finally, the findings predominantly apply to patients with severe ARDS requiring prone ventilation and may not be generalizable to other populations.

## 5 Conclusion

This meta-analysis demonstrates that EN during prone positioning in mechanically ventilated patients is associated with significantly lower caloric intake and increased risks of vomiting, high gastric residual volume, VAP, and EN interruption. While no significant difference in mortality was observed, these findings highlight the need for optimized feeding strategies to ensure nutritional adequacy and reduce complications in patients managed in the prone position. Further studies with larger sample sizes and robust designs are needed to confirm these findings and investigate interventions to improve EN tolerance and clinical outcomes in prone-positioned patients, as well as the long-term impact on recovery and post-discharge outcomes.

## Data Availability

The raw data supporting the conclusions of this article will be made available by the authors, without undue reservation.
