# Supplementary material for: Effects of prone versus supine position during enteral nutrition on clinical outcomes and complications in mechanically ventilated patients: a systematic review and meta-analysis
Source: Front Nutr. 2026 Jan 8;12:1685664. doi: 10.3389/fnut.2025.1685664 (PMC12823493; doi:10.3389/fnut.2025.1685664)
Supplement: Supplementary file 1 [file Table_1.DOCX]

Supplementary Table 1 Search Strategy for Four Databases.

| Database | Search Strategy |
| --- | --- |
| PubMed | ("Enteral Nutrition"[MeSH] OR "enteral nutrition"[tiab] OR "tube feeding"[tiab] OR "enteral feeding"[tiab] OR "gastrointestinal feeding"[tiab]) AND ("Prone Position"[MeSH] OR "prone position"[tiab] OR "ventral decubitus"[tiab] OR "prone decubitus"[tiab]) AND ("Supine Position"[MeSH] OR "supine position"[tiab] OR "dorsal decubitus"[tiab] OR "back-lying position"[tiab]) AND ("Respiration, Artificial"[MeSH] OR "mechanical ventilation"[tiab] OR "artificial ventilation"[tiab] OR "ventilated patient"[tiab] OR "intubated"[tiab]) Filters: Humans; randomized controlled trial OR cohort study; publication date ≤ June 16, 2025 |
| Embase | ('enteral nutrition'/exp OR 'enteral nutrition' OR 'tube feeding' OR 'enteral feeding' OR 'gastrointestinal feeding') AND ('prone position'/exp OR 'prone position' OR 'ventral decubitus' OR 'prone decubitus') AND ('supine position'/exp OR 'supine position' OR 'dorsal decubitus' OR 'back-lying position') AND ('mechanical ventilation'/exp OR 'mechanical ventilation' OR 'artificial ventilation' OR 'ventilated patient' OR 'intubated') AND [humans]/lim AND ([randomized controlled trial]/lim OR [cohort analysis]/lim) AND [article]/lim AND [2000–2025]/py |
| Web of Science | TS=("enteral nutrition" OR "tube feeding" OR "enteral feeding" OR "gastrointestinal feeding") AND TS=("prone position" OR "ventral decubitus" OR "prone decubitus") AND TS=("supine position" OR "dorsal decubitus" OR "back-lying position") AND TS=("mechanical ventilation" OR "artificial ventilation" OR "ventilated patient" OR "intubated") Document Types=(Article OR Review); Timespan=All years up to June 16, 2025; Language=All |
| Cochrane Library | #1 MeSH descriptor: [Enteral Nutrition] explode all trees #2 enteral nutrition:ti,ab,kw OR tube feeding:ti,ab,kw OR enteral feeding:ti,ab,kw OR gastrointestinal feeding:ti,ab,kw #3 MeSH descriptor: [Prone Position] explode all trees #4 prone position:ti,ab,kw OR ventral decubitus:ti,ab,kw OR prone decubitus:ti,ab,kw #5 MeSH descriptor: [Supine Position] explode all trees #6 supine position:ti,ab,kw OR dorsal decubitus:ti,ab,kw OR back-lying position:ti,ab,kw #7 MeSH descriptor: [Respiration, Artificial] explode all trees #8 mechanical ventilation:ti,ab,kw OR artificial ventilation:ti,ab,kw OR ventilated patient:ti,ab,kw OR intubated:ti,ab,kw #9 #1 OR #2 #10 #3 OR #4 #11 #5 OR #6 #12 #7 OR #8 #13 #9 AND #10 AND #11 AND #12 Limits: Trials and Studies; to June 16, 2025; no language restriction |
